# Reducing Insecticide Use in Broad-Acre Grains Production: An Australian Study

**DOI:** 10.1371/journal.pone.0089119

**Published:** 2014-02-19

**Authors:** Sarina Macfadyen, Darryl C. Hardie, Laura Fagan, Katia Stefanova, Kym D. Perry, Helen E. DeGraaf, Joanne Holloway, Helen Spafford, Paul A. Umina

**Affiliations:** 1 CSIRO Ecosystem Sciences and Sustainable Agriculture Flagship, Canberra, Australia; 2 Department of Agriculture and Food, Irrigated Agriculture and Diversification, Perth, Australia; 3 The University of Western Australia, School of Animal Biology, Perth, Australia; 4 The University of Western Australia, The UWA Institute of Agriculture, Perth, Australia; 5 South Australian Research and Development Institute, Entomology Unit, Urrbrae, Australia; 6 New South Wales Department of Primary Industries, Wagga Wagga Agricultural Institute, Wagga Wagga, Australia; 7 The University of Melbourne, Department of Zoology, Melbourne, Australia; 8 cesar, Melbourne, VIC, Australia; French National Institute for Agricultural Research (INRA), France

## Abstract

Prophylactic use of broad-spectrum insecticides is a common feature of broad-acre grains production systems around the world. Efforts to reduce pesticide use in these systems have the potential to deliver environmental benefits to large areas of agricultural land. However, research and extension initiatives aimed at decoupling pest management decisions from the simple act of applying a cheap insecticide have languished. This places farmers in a vulnerable position of high reliance on a few products that may lose their efficacy due to pests developing resistance, or be lost from use due to regulatory changes. The first step towards developing Integrated Pest Management (IPM) strategies involves an increased efficiency of pesticide inputs. Especially challenging is an understanding of when and where an insecticide application can be withheld without risking yield loss. Here, we quantify the effect of different pest management strategies on the abundance of pest and beneficial arthropods, crop damage and yield, across five sites that span the diversity of contexts in which grains crops are grown in southern Australia. Our results show that while greater insecticide use did reduce the abundance of many pests, this was not coupled with higher yields. Feeding damage by arthropod pests was seen in plots with lower insecticide use but this did not translate into yield losses. For canola, we found that plots that used insecticide seed treatments were most likely to deliver a yield benefit; however other insecticides appear to be unnecessary and economically costly. When considering wheat, none of the insecticide inputs provided an economically justifiable yield gain. These results indicate that there are opportunities for Australian grain growers to reduce insecticide inputs without risking yield loss in some seasons. We see this as the critical first step towards developing IPM practices that will be widely adopted across intensive production systems.

## Introduction

There are a range of management practices associated with the production of broad-acre grain crops, including the use of modern crop varieties, irrigation, fertiliser, and crop protectants to control losses from arthropod pests, disease and weeds. The availability and widespread use of agricultural pesticides since the 1950′s is one factor that has enabled farmers to produce increasing yields of high quality food. However, these practices, either individually or cumulatively, have contributed to a substantial loss of biodiversity in agricultural landscapes around the world. Geiger *et al.* [1] assessed 13 components of intensification in European farmland and found that the use of insecticides and fungicides had consistent negative effects on biodiversity. This realisation has fuelled a policy debate around the use of pesticides, resulting in the loss of pesticides in some countries due to regulatory reviews, and the introduction of legislation that mandates low pesticide-input farming in the European Union [2]. It is highly likely that there will be fewer pesticides available to farmers in the future and those that are available will be selective, more expensive, and will need to be used more strategically.

**Figure 1:**
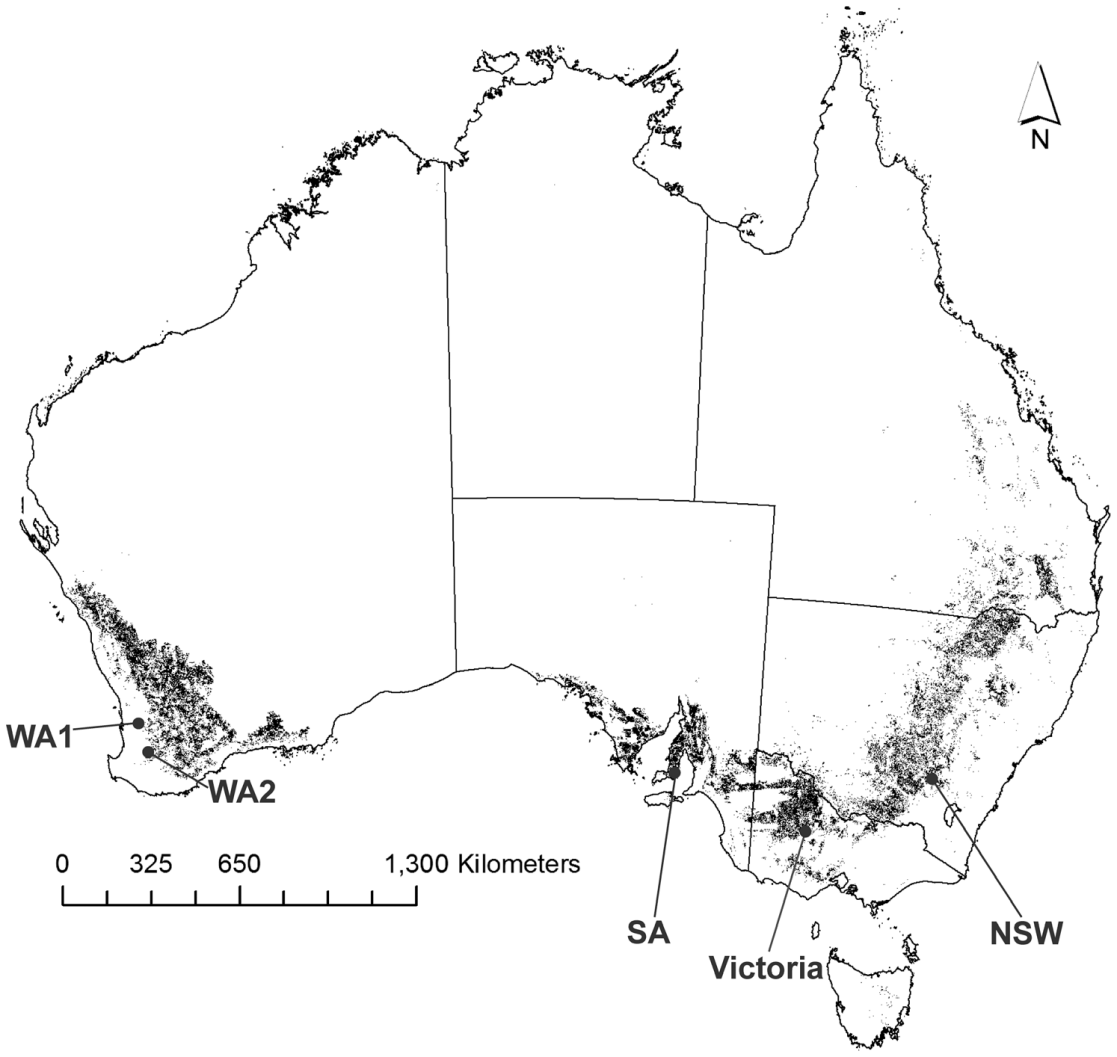
Map showing the location of trial sites throughout grain growing regions of southern Australia. Black shaded areas show where broad-acre cereals and oilseeds are grown. At each site large plots (50 m×50 m minimum) with three pest management approaches were assessed. Trials were conducted on canola in 2010 and wheat in 2011. Land use data comes from ABARES Land Use of Australia, Version 4, 2005/2006 (September 2010 release).

Australia is one of the larger grain producing countries in the world. Grain crops are grown primarily in dryland conditions in a large arc around the continent under a wide range of climates (Fig. 1). Wheat and barley account for 74% of total arable crop sowings and other crops such as canola, lupins, oats, sorghum and cotton are grown in smaller areas [3]. Grain crops are attacked by a diversity of arthropod pest species whose populations can be highly sporadic across space and time. Furthermore, the importance of particular pests has changed over recent years, with some becoming more problematic and others less so [4]. Farming practices that may have driven this change include the transition to minimum or no-tillage systems, changes to weed management, the introduction of GM cotton that expresses an insecticide, a significant increase in the total area sown to canola, and continued reliance on pesticides leading to resistance in some pest species [4], [5]. Insecticide resistance has been recorded for several important arthropod pests, including the green peach aphid (*Myzus persicae*) [6], redlegged earth mite (*Halotydeus destructor*) [7], diamond back moth (*Plutella xylostella*) [8], *Helicoverpa* spp. [9], and the Western flower thrips (*Frankliniella occidentalis*). Several others species, such as *Balaustium medicagoense*, *Bryobia* sp. and *Sminthurus viridis* have a high natural tolerance to some insecticides [10], [11]. How these pests can be effectively controlled in the future, under new cropping systems, without unacceptably high environmental costs, needs to be determined.

IPM has been the archetype model for controlling pests in a sustainable manner for over 50 years [12]. We have evidence that IPM can work in many farming systems and can reduce over-reliance on broad-spectrum insecticides. Pretty [13] examined 62 IPM initiatives in 26 countries and found a reduction in pesticide-use over time in the majority of these cases (around 50% reduction on average). A study using 539 wheat fields in Germany found that fields that used some IPM strategies had lower pesticide use [14]. In certain crops, IPM can also lead to economic savings. For example, Reddy [15] calculated that a lower-input IPM strategy, that relied on biocontrol agents in cabbage, was almost US$100 per ha cheaper than a conventional pest control approach. In theory, IPM involves the use of cultural, biological and chemical control techniques with a full understanding of the relationship between pest ecology and abundance, plant damage and yield loss [16]. IPM requires a strong understanding of how beneficial and pests interact, move around the landscape, and use resources outside the field and between cropping seasons [17], [18]. Insecticides may be used as part of an IPM strategy, however, in principle should only be applied as a last resort, after pest populations have reached a threshold, beyond which economic yield losses will be incurred. This threshold approach is fundamental to IPM practice.

Despite the longevity of IPM, the majority of grain growers in Australia continue to rely heavily on the use of cheap broad-spectrum insecticides to control pests [19]. IPM is more knowledge-intensive than a conventional approach that relies primarily on prophylactic applications of insecticides [20]. Monitoring to determine pest abundance takes time. Some checks for pest species in crops are conducted prior to spraying, but there are typically few regular scouting activities throughout the season. Furthermore, even if economic thresholds have been clearly defined they are not always adhered to for a variety of reasons [21]. Very few selective insecticides are available to growers, and those that are available are relatively expensive [19], [22]. Often IPM principles are not consistently implemented and true ‘integration’ of multiple control techniques is uncommon [13], [22].

The aim of this study was to quantify the impact of different pest management approaches on arthropod pests and beneficial arthropods in grain crops grown across southern Australia. We test the hypothesis that a high levels of insecticide use (as seen in conventional practices) results in fewer pests in grain crops, less crop damage from pests, and no yield loss. We contrast the conventional practice with a low-input approach that uses monitoring of pest abundance to decide if an insecticide application is necessary. We test this hypothesis using replicated trials set in commercial fields, across five sites that span the diversity of contexts in which grain crops are grown, and across two cropping seasons. This implies our results can be used to highlight the situations when growers could reduce pesticide inputs without impacting on profitability. We see this as a critical first step towards developing sustainable pest management practices, like IPM, that can be widely adopted by Australian growers in broad-acre production systems.

## Materials and Methods

### Ethics Statement

All research was carried out on private properties. Permission to be on the land and conduct the research was given by the landholders. Sampling did not involve endangered or protected species. Data sets relating to the analysis presented here can be found at [23].

### Description of Trial Sites

Five on-farm sites (labelled as Victoria, NSW, SA, WA1, and WA2 throughout) were established using a standardised experimental design and sampling protocols across the dryland grain growing regions of Australia (Fig. 1). Trials were undertaken in collaboration with a local farming-systems group and the landholder at each site. At three sites (Victoria, WA1 and WA2) these were long-term perennial pasture paddocks, while the previous years’ crops were barley and lentils at the NSW and SA sites, respectively. In 2010, trials were established at each site using canola. In 2011, the trials were repeated using the same field plots, but with wheat. Each trial consisted of three comparative pest management strategies: a control (ideally no insecticides applied), a conventional “high-input” pest management approach (based on use of preventative and remedial insecticides currently used by many growers within the local region), and an alternative “low-input” pest management approach (where scouting information was used to decide when to apply an insecticide and, if possible, choice of a softer chemical option). Full insecticide details are listed in Table 1. All other chemical inputs (i.e. fertiliser, herbicide) and farming practices were reflective of the practices used by the landholder at each site. A randomised complete block design with 12 plots arranged in a three by four matrix was used across all sites. There were four replicate plots per treatment. Plot size varied across sites ranging from a minimum of 50 m by 50 m to 75 m by 75 m (up to 5625 m^2^ per plot) depending on local seeding equipment. The plots were positioned within a larger field of the same crop and cultivar. At each site, plots were established 10–50 m from the nearest field edge. In some instances, the use of seed treated with insecticide could only be used across all plots due to the seeding practices being employed.

**Table 1:** Summary of insecticide inputs applied to each trial site across Australia.

Treatment	Crop (cultivar)	Insecticide seed treatment	Insecticide foliar treatment$
**2010– Victoria (350 mm)**			
Control	Canola (Clearfield 44C79)	–	–
Conventional	Canola (Clearfield 44C79)	–	alpha-cypermethrin (PSPE); omethoate (PE)
Low input	Canola (Clearfield 44C79)	imidacloprid	–
**2011– Victoria (184 mm)**			
Control	Wheat (Correll)	–	–
Conventional	Wheat (Correll)	–	alpha-cypermethrin (PE)
Low input	Wheat (Correll)	–	–
**2010– NSW (337 mm)**			
Control	Canola (Hybrid 46Y78)	imidacloprid	–
Conventional	Canola (Hybrid 46Y78)	imidacloprid	bifenthrin (PSPE); omethoate (PE)
Low input	Canola (Hybrid 46Y78)	imidacloprid	–
**2011– NSW (203 mm)**			
Control	Wheat (Sunvale)	–	–
Conventional	Wheat (Sunvale)	–	bifenthrin (PSPE)
Low input	Wheat (Sunvale)	–	–
**2010– SA (406 mm)**			
Control	Canola (Hybrid 46Y78)	imidacloprid	–
Conventional	Canola (Hybrid 46Y78)	imidacloprid	dimethoate+bifenthrin (PE)
Low input	Canola (Hybrid 46Y78)	imidacloprid	–
**2011– SA (238 mm)**			
Control#	Wheat (Mace)	–	–
Conventional#	Wheat (Mace)	–	omethoate+alpha-cypermethrin (PE)
Low input #	Wheat (Mace)	imidacloprid	–
**2010 - WA1 (144 mm)**			
Control	Canola (Argyle)	–	–
Conventional	Canola (Argyle)	–	bifenthrin+chlorpyrifos (PSPE); chlorpyrifos+dimethoate (PE)
Low input	Canola (Argyle)	–	dimethoate (PE); pirimicarb +*Bt* (LS)
**2011 - WA1 (376 mm)**			
Control	Wheat (Magenta)	imidacloprid	–
Conventional	Wheat (Magenta)	–	alpha-cypermethrin+chlorpyrifos (PS); alpha-cypermethrin (LS)
Low input	Wheat (Magenta)	–	–
**2010– WA2 (139 mm)**			
Control	Canola (Cobbler)	–	–
Conventional	Canola (Cobbler)	–	chlorpyrifos (PS); bifenthrin (PE)
Low input	Canola (Cobbler)	–	–
**2011– WA2 (341 mm)**			
Control	Wheat (Bullaring)	–	–
Conventional	Wheat (Bullaring)	–	cypermethrin (LS)
Low input	Wheat (Bullaring)	–	–

Growing season rainfall is shown in brackets. In 2010 the crop was canola and in the same location, wheat in 2011.

$ PS = pre-sow; PSPE = post-sowing, pre-emergence; PE = post-emergence; LS = late season foliar treatments.

# An aerial application of metaldehyde was used across all plots to control snails late season.

### Arthropod Sampling

We used three sampling techniques to collect arthropods throughout the season; vacuum samples (to collect foliage and litter dwelling arthropods early in the season), sweep nets (to collect foliage dwelling arthropods late in the season) and pitfall traps (to collect ground dwelling, and night active arthropods). These sampling techniques are able to capture a range of arthropod pest and beneficial species in broad-acre crops, and are easily replicable across sites, however each will be more efficient at capturing some species than others [24], [25]. All sampling was conducted at least 10 m from the edge of plots to account for edge effects caused by the movement of arthropods between plots. Vacuum sampling was conducted prior to crop sowing and at approximately 7, 14, 28 and 42 days after crop emergence (DAE) depending on the prevailing weather conditions. A modified, petrol-driven, leaf blower with a plastic tube inserted over the fan was used to collect the arthropods off the crop, other vegetation and soil surface. A bag or 100-micron fine cup sieve was fitted on to the end of the vacuum spout to capture arthropods. A rectangular frame (150 mm×600 mm) was placed on the soil surface over a row of plants and the nozzle of the suction sampler moved over the soil surface and plant material in this area for 5–10 seconds. A minimum of five samples and a maximum of 10 replicate samples were taken within each plot. Samples were taken at random locations within the plot at each time point (but usually no closer than 10 m from each other).

Sweep samples were used when the crop became too tall for vacuum sampling, generally from flowering to grain ripening. The total number of sampling points at each site varied depending on the crop development stage and weather conditions at the time of sampling. The sweep net consisted of a 380 mm diameter rigid aluminium hoop with a fine mesh net attached. Each sample consisted of 6 or 10 sweeps in canola (a single sweep was a 180° arc covering approximately 2 m with one stride per sweep) in five locations in each plot. In the wheat in 2011 each sample consisted of six sweeps. The sample contents were transferred to a plastic bag or a vial containing 70% (v/v) ethanol.

Pitfall sampling was conducted at several times throughout the growing season. The first sample was taken prior to crop emergence, with additional samples during crop establishment and spring. Each trap consisted of a polyvinyl chloride (PVC) sleeve that was placed in the ground (flush with the ground surface) at the start of the season using a solid steel pin or by excavating holes using a trowel depending on soil type. Vials (45 mm diameter, 120 mL volume) containing 30–60 mL of a propylene glycol (50%): water (50%) mixture, were placed inside the sleeves and left open for seven days. After this time the traps were collected and a lid placed over the sleeve until the next sampling interval. In NSW, SA, WA1 and WA2 nine pitfall traps, arranged in a 3×3 grid, were placed in each plot. However the numbers sorted ranged from three to nine traps per plot and were randomly chosen from those that had been sampled. In SA five traps were sorted per plot (the four outside corners of the grid and a central trap). In Victoria five pitfall traps were placed in a regular arrangement in the central 10 m x 10 m area of each plot. Four traps were placed in a square configuration, 5 m apart from each other, and the fifth placed centrally. All five traps were sorted from the Victorian site.

Direct visual observations of arthropod abundance were also made regularly throughout the season. This information was used to determine if an economic threshold had been reached in the low input treatment plots and therefore an insecticide application was necessary. This involved walking through the plots (from 3 starting positions) and examining sections that had missing plants, uneven plant growth, and ‘hotspots’ of chewing and/or sucking damage caused by pest feeding. If such an area occurred, the abundance of arthropod pests was determined using quadrat counts on the soil and plants in autumn and winter, or searching plants and taking sweep net samples in spring.

### Arthropod Sorting and Identification

Samples were returned to the laboratory for sorting under a stereomicroscope. We did not identify all arthropods collected, but focussed on identifying key pest and beneficial species in each system. These species are known to cause damage to grain crops in southern Australia or are known to attack arthropod pests of grain crops. Other species were classified down to Family level where possible. Taxa were categorised into three groups: pest arthropods, beneficial arthropods, and other arthropods. Other arthropods were excluded from the analyses presented here. Examples of which taxa were included in each of these groups can be found in the supplementary material (Table S1). In WA pitfall traps all Collembola and Acari (mites) were excluded from the sorting owing to the extremely large numbers of these organic recyclers present in many of the samples.

### Yield and Harvest Index Estimates

At all sites crop yield was estimated in each plot at the end of the cropping season using harvesting machines suitable for small-plots. The approach to estimate yield differed across sites. In Victoria there were three harvester passes (wheat approx. 80 m^2^ and canola approx. 150 m^2^ per plot) within the 30 m by 30 m centre area of each plot. In NSW canola and wheat, the harvester cut a single swath of 1.85 m wide by 75 m long in each plot (138.75 m^2^ area per plot). In SA wheat, the harvester cut three swaths per plot of 1.8 m wide by 10 m long (54 m^2^ area per plot). In SA canola a yield map of the entire plot was constructed using a GPS Trimble RTK system with 2 m accuracy linked to the farmer’s harvester taking a reading every two seconds. A single “sample” per plot was estimated from this data by averaging all recorded yield points within the plot area. In WA1 canola and wheat the harvester cut five swaths of 10 m wide by 70 m long in each plot (3500 m^2^ area per plot). In WA2 canola the harvester cut five swaths of 1.25 m wide by 70 m long in each plot (437.5 m^2^ area per plot). In 2011, WA2 wheat, no yield data was collected.

At the end of the season, Harvest Index (HI) was estimated by hand-cutting and drying plants. At 6–10 locations in the 30 m by 30 m centre area of each plot a stick was placed along the ground (usually 1 m in length) and all plants cut at ground level. The plants were put into paper bags and allowed to air dry for at least seven days except in Western Australia where they were oven dried. For canola, once dried to <8% seed moisture content the pods were threshed to separate out the seed and all the seeds weighed. The remaining plant material was also weighed after drying. HI was calculated as a proportion of total seed weight by total plant biomass for each sampling location. The same was performed in 2011 for wheat. In WA2 canola, HI was not assessed.

### Plant Assessments

All plant assessments were conducted at least 10 m from the plot edge. An assessment of feeding damage from arthropod pests on plants was conducted at 7, 14, 28, and 42 DAE. A maximum of 10 samples were taken at random locations within each plot (and usually no closer than 10 m from each other). At each sample location, a stick (usually 1 m in length) was placed on the ground along a row of plants, and the total number of plants counted. Row spacing was recorded so that plant density (per m^2^) could be calculated. In SA the length sampled was adjusted based on row spacing to give a total sample area of 2.5 m^2^ per plot (after 10 samples). The number of plants along a stick with chewing damage (Chew damage) and sucking damage (Suck damage) were recorded at each sample location. An overall feeding severity score was measured for the damaged plants along the stick, based on a 0–10 scale. Zero indicates no visible damage, five indicates approximately 50% leaf area damaged, averaged across all plants, and 10 indicates all plants dead or dying. This score has been validated by numerous authors working on grain pest arthropods [26], [27]–[29]. The overall damage at each sample location was expressed as a proportion out of 10 using the formula:
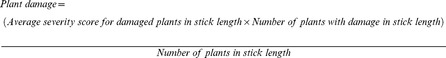



We also recorded the amount of crop cover and amount of weed cover at each location within the plot as an overall percentage.

### Statistical Models and Analyses

Generalized linear mixed models [30] were used for the analyses of plant damage and arthropod count data. Due to the presence of DAE explanatory variable and consequently possible inclusion of polynomials of DAE in models, it is more precise to describe the fitted models as generalised additive mixed models (GAMMs). For all response variables presented as proportions, a binomial distribution was assumed and the link function used was the canonical link – logit. Similarly, all responses presented as counts were assumed Poisson distributed and the logarithmic (log) link function was used. The ratio of deviance (χ^2^ approximation of residual deviance) to the degrees of freedom, called variance inflation factor *c*, was used to assess over-dispersion. The model selection aimed at getting an adequate fit without over-fitting and was, in general, based on Akaike Information Criterion (AIC or AIC_c_ for small samples). In cases of over-dispersion the QAIC and Q AIC_c_ [31] were used, respectively. For all trials the following response variables were analysed using the above explained statistical models: plant density, sucking damage, chewing damage, pest abundance, and beneficial abundance. In all models, Treatment factor (representing the three pest management approaches) and DAE were fitted as fixed, along with Treatment interactions with DAE and polynomials of DAE up to third degree. The blocking/plot structure was accounted for in the random part of the model. For the majority of fitted models the over-dispersion was due to outliers or the fit of covariates and was corrected. In the cases where the over-dispersion was due to the nature of the data (e.g. too many zeros or clustering of the data), the ASReml-R option for over-dispersion was used.

Yield and HI were analysed using linear mixed models (LMM) formulated using a randomization-model based approach. Typically, the model for each trait and trial included blocking terms to account for the randomization process, and additional terms to model the treatment effects, the covariates and the extra sources of variation, such as spatial trends and extraneous variation. Our methodology was based on the approach of Gilmour *et al.* [32], followed by additional diagnostics to assess the adequacy of the spatial model [33]. The initial mixed model for each trait by trial comprised random replicate, fixed treatment effect and a separable (column by row) autoregressive process of first order to account for the local spatial trend. After fitting this model, the residuals were checked (residual plots, variogram and faces of the variogram with 95% coverage intervals) to model additional spatial variation (global trend) and/or extraneous variation. We adopted this approach only for the SA canola yield, where the full spatial configuration of the trial was present. The analysis of the Victoria canola yield included a covariate to account for the percentage of lodging affected area and the angle of lodging for each sample. The significance of the fixed terms in the model, in this case the Treatment term, was assessed using Wald test statistic, which has an asymptotic chi-squared distribution (χ^2^) with degrees of freedom equal to those of the treatment term.

Additionally, yield estimates from each trial were combined and statistical techniques for the analysis of a multi-environment trial (MET) were used to compare the different types of insecticide inputs used in each pest management approach. In this case the treatments were defined as a combination of foliar treatments during the early and late season, snail baits and insecticidal seed treatments. Treatment levels used in the MET analysis for canola were: ES for early season spray, ES_FS for combined early and late season sprays, ES_S for combined early season spray with seed treatment, S for seed treatment only, and N for no insecticide inputs. The combination of treatments applied to wheat trials were: ES for early season spray, ES_FS for combined early and late season sprays, S for seed treatment only, SB for snail baits, SB_ES for snail baits and early season spray, SB_S for snail baits and seed treatment, and N for no insecticide inputs. The data were analysed using ASReml-R [34], which facilitates joint modelling of blocking, treatment structure, spatial and extraneous terms and accommodates MET analyses.

Economic costs were calculated to estimate the price of insecticide inputs across each treatment at the different trial sites. This was performed using input prices (in Australian dollars) derived from chemical re-sellers in Victoria, Australia (current as of February 2013). Application costs, which assume all insecticides were applied via ground-rig, were included in the total economic price for each treatment.

## Results

Using the three sampling techniques we collected large numbers of arthropods from a range of species (298,869 individuals, Table S2). Across the two years of the study pest pressure was generally low, and only one site reached established threshold levels for crop pests (WA1 canola late in the season suffered significant aphid attack). However, we still collected large numbers of pests (118,393 in canola, 116,614 in wheat, Table S2) and found variability in the numbers of pests at each trial site and within each plot. The low pest pressure led to very few insecticide applications on the low input plots, and, in seven out of the 10 trials, the insecticides applied to the low input plots were the same as for the control plots (Table 1). The sites in WA experienced drought conditions in 2010, with rainfall well below the growing season average. We summarise our results below by firstly highlighting the hypothesised pattern and then comparing this to the data collected in each trial. For those traits in which we found a significant treatment effect (overall *P*-value for treatment <0.05, and/or interaction between treatment and DAE <0.05) we have ranked the multiple comparison results for each trait (using the standard error of the difference). For example, “control>LI>conventional” indicates that for this trait, on average, the control plots had the greatest values, next the low input plots, and lastly the conventional plots. A bracket around two treatments, e.g. (control/LI)>conventional, indicates that there was no significant difference between these two treatments. Multiple brackets around all treatments, e.g. (control/(conventional)/LI), indicate a significant difference only between the highest and lowest treatments.

### Impact of the Pest Management Approach on Pest and Beneficial Arthropods

We hypothesised that there should be lower abundance of pest and beneficial arthropods in plots that received greater insecticide inputs (control>LI>conventional). Overall we did find many significant effects of pest management approach on pest abundance across all trials and sampling techniques (Table 2). Often this was not consistent across the time period as evidenced by a significant interaction between treatment and time (Table 2). For pest abundance in canola, out of the 15 possible combinations of site by sampling technique, seven showed significant effects of treatment and 12 showed significant interactions between treatment and time. Given the greater frequency of early-season insecticide applications across the trials we would expect the vacuum samples to show the clearest response to treatment (Figure C in File S1, Figure C in File S2, Figure C in File S3, Figure C in File S4, and Figure C in File S5). Four out of five canola trials showed a significant effect of treatment on pest abundance in vacuum samples that matched our expectations of greater pest abundance in the control or low input plots. The NSW canola trial (Figure C in File S2) is a good example of this pattern, with decreased pest abundance across time in the conventional plots. In the wheat trials, eight models showed significant effects of treatment, and 10 showed significant interactions between treatment and time (Table 2). Only one trial (WA1), showed a non-significant effect of treatment on pest abundance in wheat vacuum samples. Only two of the five trials matched our expectation of greater pest abundance in the control or low input plots.

**Table 2:** The effect of different pest management approaches on pest arthropod abundance.

Site	Samplingtechnique	TreatmentP-value^$^	treatment×DAE interactionP-value^$^	Ranking#
**Canola**				
Victoria	Pitfall	0.0044**	<0.001***	Control>LI>conven ^B^
NSW	Pitfall	<0.001***	<0.001***	Conven>control>LI ^A^
SA	Pitfall	0.78	<0.001***	LI>control>conven ^A^
WA1	Pitfall	0.41	0.027*	(Control/LI/conven) ^C^
WA2	Pitfall	0.24	<0.001***	(Control/LI/conven) ^C^
Victoria	Vacuum	<0.001***	<0.001***	(Control/LI)>conven ^B^
NSW	Vacuum	<0.001***	0.0026**	(LI/control)>conven ^B^
SA	Vacuum	0.0058**	0.010*	(LI/control)>conven ^B^
WA1	Vacuum	0.020*	<0.001***	Control>LI>conven ^B^
WA2	Vacuum	0.48	0.065	NP^ C^
Victoria	Sweep	0.012*	<0.001***	Control>(conven/LI)^ A^
NSW	Sweep	0.70	0.69	NP^ C^
SA	Sweep	0.83	0.56	NP^ C^
WA1	Sweep	0.092	<0.001***	Conven>(LI/control)^ A^
WA2	Sweep	0.44	<0.001***	Control>(LI/conven)^ A^
**Wheat**				
Victoria	Pitfall	0.035*	<0.001***	(Control/LI)>conven ^B^
NSW	Pitfall	0.15	<0.001***	Conven>(control/LI)^ A^
SA	Pitfall	0.15	<0.001***	(Control/conven)>LI^ A^
WA1	Pitfall	0.31	0.37	NP^ C^
WA2	Pitfall	0.0017**	0.26	Conven>LI>control^ A^
Victoria	Vacuum	0.0039**	<0.001***	(LI/control)>conven^ B^
NSW	Vacuum	<0.001***	0.0074**	Control>LI>conven ^B^
SA	Vacuum	0.047*	<0.001***	Control>(conven/LI)^ A^
WA1	Vacuum	0.56	0.0015**	Conven>(LI/control)^ A^
WA2	Vacuum	0.0094**	0.049*	(LI/(control)/conven)^ A^
Victoria	Sweep	0.79	0.80	NP^ C^
NSW	Sweep	<0.001***	–	(LI/control)>conven ^B^
SA	Sweep	<0.001***	0.10	Control>LI>conven ^B^
WA1	Sweep	0.090	<0.001***	(Control/LI)>conven ^B^
WA2	Sweep	0.38	<0.001***	(LI/(control)/conven)^ A^

A GAMM analysis was used to assess the effect of three pest management approaches (treatment: conventional, low input (LI), or control) and time (DAE, days after crop emergence) on the abundance of all arthropod pests collected using three different sampling techniques.

# A, a significant difference between treatments but the pattern does not follow what we expect; B, a significant difference between treatments and abundance was highest in control (or low input) and lowest in the conventional (control>LI>conven); C, no difference in pest abundance between the treatments. In this case no ranking was provided (NP). ^$^ P-value of *<0.05, ** <0.01, *** <0.001.

For beneficial arthropod abundance, there were fewer significant effects relating to pest management approach (Table 3). In the canola trials, three models showed significant effects of treatment, and seven showed significant interactions between treatment and time. These significant results were seen for the ground-dwelling species collected using pitfall traps and species collected from the plant foliage using the vacuum sampler. In wheat trials, three models showed significant effects of treatment, and five showed significant interactions between treatment and time (Table 3). The pest and beneficial abundance at one site (Victoria, canola) in vacuum samples (Figure C in File S1) most clearly supported our hypothesis with conventional plots having the lowest numbers of both pest and beneficial arthropods at multiple sample dates. Additional graphs showing the pest and beneficial abundance in each trial across time using pitfalls and sweep net sampling can be found in Figures A & B in File S1, Figures A & B in File S2, Figures A & B in File S3, Figures A & B in File S4, and Figures A & B in File S5.

**Table 3:** The effect of different pest management approaches on beneficial arthropod abundance.

Site	Samplingtechnique	Treatment*P*-value^$^	treatment×DAEinteraction *P*-value^$^	Ranking#
**Canola**				
Victoria	Pitfall	<0.001***	<0.001***	(Control/LI)>conven^B^
NSW	Pitfall	0.041*	<0.001***	(LI/control)>conven^B^
SA	Pitfall	0.43	<0.001***	(LI/(conven)/control)^A^
WA1	Pitfall	0.50	<0.001***	(Control/(LI)/conven)^ A^
WA2	Pitfall	0.24	0.41	NP^ C^
Victoria	Vacuum	0.0011**	0.23	(LI/control)>conven^B^
NSW	Vacuum	0.14	0.022*	(LI/control)>conven^B^
SA	Vacuum	0.82	0.0025**	(Conven/(control)/LI)^ A^
WA1	Vacuum	0.38	0.55	NP^ C^
WA2	Vacuum	0.81	0.68	NP^ C^
Victoria	Sweep	0.19	0.31	NP^ C^
NSW	Sweep	0.70	0.11	NP^ C^
SA	Sweep	0.90	0.019*	(Conven/(LI)/control)^ A^
WA1	Sweep	0.35	0.098	NP^ C^
WA2	Sweep	0.32	0.89	NP^ C^
**Wheat**				
Victoria	Pitfall	0.94	0.053	NP^ C^
NSW	Pitfall	0.68	<0.001***	(LI/(conven)/control)^ A^
SA	Pitfall	0.21	<0.001***	(Control/LI)>conven^B^
WA1	Pitfall	0.90	0.85	NP^ C^
WA2	Pitfall	0.40	0.067	NP^ C^
Victoria	Vacuum	0.96	0.0036**	(Control/(conven)/LI)^ A^
NSW	Vacuum	0.0031**	0.71	(Control/(LI)/conven)^ A^
SA	Vacuum	0.012*	<0.001***	(Control/(conven)/LI)^ A^
WA1	Vacuum	0.78	0.53	NP^ C^
WA2	Vacuum	0.52	0.78	NP^ C^
Victoria	Sweep	0.48	0.43	NP^ C^
NSW	Sweep	0.72	0.64	NP^ C^
SA	Sweep	0.42	0.014*	(Conven/(control)/LI)^ A^
WA1	Sweep	0.082	0.61	NP^ C^
WA2	Sweep	<0.001***	0.52	(LI/control)>conven^B^

A GAMM analysis was used to assess the effect of three pest management approaches (treatment: conventional, low input (LI), or control) and time (DAE, days after crop emergence) on the abundance of all beneficial arthropods (predators and parasitoids) collected using three different sampling techniques.

# A, a significant difference between treatments but the pattern does not follow what we expect; B, a significant difference between treatments and abundance was highest in control (or low input) and lowest in the conventional (control>LI>conven); C, no difference in pest abundance between the treatments. In this case no ranking was provided (NP). ^$^ P-value of *<0.05, ** <0.01, *** <0.001.

### Impact of the Pest Management Approach on Crop Plant Damage

We hypothesised that there should be lower levels of crop plant damage from arthropod pests (control>LI>conventional) and higher plant density (conventional>LI>control) in plots that received greater insecticide inputs. We found that whilst there were some significant effects of pest management approach on plant damage estimates (Table 4), overall the amount of plant damage was relatively low. Chewing damage was more prevalent than sucking damage, but only at one site did we see very high levels of chewing damage (over 35% at WA1 canola). The pattern of plant damage generally supported our hypothesis in the canola trials, with control plots having the greatest relative amount of chewing damage (Table 4). However, in wheat our hypothesis was not supported with many trials showing similar levels of plant damage across the three pest management approaches. We measured plant density to account for plants that were completely removed at the early growth stage by arthropod pests. The pest management approach used had a significant impact on plant density in the Victoria, WA1 and WA2 sites with canola and all wheat trials (Table 4). However, the patterns between treatments did not always match the hypothesis. For example, in only two canola trials did the conventional plots have higher plant density, suggesting that more plants had been damaged by the activities of arthropod pests in the control and low input plots.

**Table 4:** The effect of pest management approach on estimates of crop plant damage.

Site	Treatment *P*-value^$^	treatment×DAEinteraction *P*-value^$^	Ranking#
**Sucking damage**			
**Canola**			
Victoria	<0.001***	<0.001***	Control>LI>conven^B^
NSW	0.071	0.75	NP^ C^
SA	–	–	–
WA1	<0.001***	0.69	(Control/(LI)/conven)^ A^
WA2	0.78	0.96	NP^ C^
**Wheat**			
Victoria	0.36	0.020*	Control>(conven/LI)^ A^
NSW	0.013*	0.088	LI>(control/conven)^ A^
SA	–	–	–
WA1	0.74	0.78	NP^ C^
WA2	0.92	0.71	NP^ C^
**Chewing damage**			
**Canola**			
Victoria	<0.001***	<0.001***	Control>(LI/conven)^ B^
NSW	0.0017**	0.54	(Control/LI)>conven^B^
SA	0.023*	0.93	(Control/(LI)/conven)^ B^
WA1	0.17	0.88	NP^ C^
WA2	0.0017**	0.69	(Control/LI)>conven^B^
**Wheat**			
Victoria	NA	NA	NA
NSW	0.47	<0.001***	(Conven/(LI)/control)^ A^
SA	<0.001***	0.77	Control>conven>LI^A^
WA1	0.21	0.93	NP^ C^
WA2	0.20	0.78	NP^ C^
**Plant density**			
**Canola**			
Victoria	<0.001***	<0.001***	LI>conven>control^A^
NSW	0.095	0.0051**	LI>(control/conven)^ A^
SA	0.27	<0.001***	Control>(LI/conven)^ A^
WA1	<0.001***	<0.001***	Conven>(LI/control)^ B^
WA2	<0.001***	0.045*	Conven>control>LI^B^
**Wheat**			
Victoria	<0.001***	<0.001***	Control>conven>LI^A^
NSW	0.0029**	0.37	Control>(LI/conven)^ A^
SA	<0.001***	<0.001***	LI>conven>control^A^
WA1	<0.001***	<0.001***	(Conven/control)>LI^A^
WA2	<0.001***	0.44	Control>(conven/LI)^ A^

A GAMM analysis was used to assess the effect of three pest management approaches (treatment: conventional, low input (LI), or control) and time (DAE) on plant damage from feeding by pest herbivores. A dash indicates that data was not collected during that trial and NA indicates that a model couldn’t be fitted due to zeros in data set.

# A, a significant difference between treatments but the pattern does not follow what we expect; B, a significant difference between treatments and damage was highest in control (or low input) and lowest in the conventional (control>LI>conven), or for plant density we expect greatest density in the conventional and lowest in the control (or low input) (conven>LI>control); C, no difference in plant damage or density between the treatments. In this case no ranking was provided (NP). ^$^ P-value of *<0.05, ** <0.01, *** <0.001.

### Impact of the Pest Management Approach on Crop Yield

**Figure 2:**
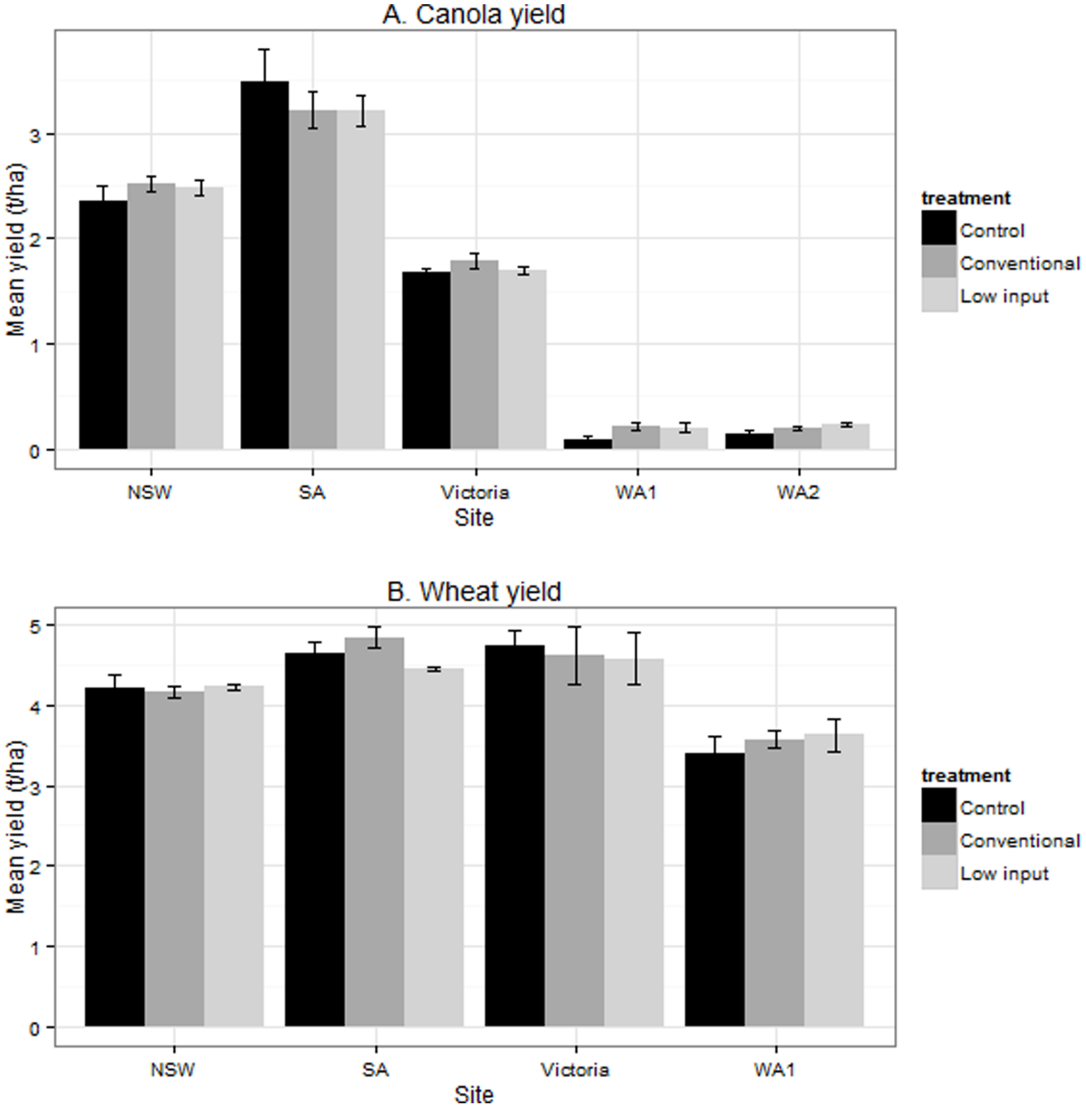
Impact of pest management approach on crop yield in small-plot trials of canola (A) in 2010 and wheat (B) in 2011. Trials were conducted at five sites across the grain growing regions of Australia. There were three pest management approaches assessed (conventional, low input, or control). Overall we found no significant effect of pest management approach on crop yield. In SA wheat (B) we found a significant effect but this was sensitive to the presence or absence of one sample point. In WA1 canola there was a marginally significant effect on yield (*P* = 0.049, (conventional/LI)>control). Bars indicate the mean of 4 replicate plots and 1×SE.

We hypothesised there would be higher crop yield in plots that received greater insecticide inputs due to less damage from arthropod pests (conventional>LI>control). The results from the analyses typically showed no significant treatment effects (Fig. 2) in relation to yield (8 out of 10 trials). In WA1 canola there was a marginally significant effect on yield (*P* = 0.049, (conventional/LI)>control). There was a significant difference in yield in the SA wheat trial, however this result was sensitive to the addition or removal of one sample point (with outlier *P* = 0.116; outlier deleted *P* = 0.003, conventional>(control/LI)). An estimate of HI was made at the end of the season in each plot to examine the ratio of grain yield to plant biomass. We found less consistent effects of pest management approach on HI. Two out of four canola trials showed a significant effect (Victoria *P* = 0.001 control>(conventional/LI), WA1 *P* = 0.034 (LI/conventional)>control)). Of the five wheat trials, only WA1 showed significant differences in relation to pest management approach (*P* = 0.012, (LI/conventional)>control).

Due to the method used to harvest the canola in SA, yield was analysed using a spatial linear mixed model. The aim of the spatial analysis is to adjust for the natural variation (by fitting autocorrelations for the local trend and regressions on row/column number for the global row/column trends, respectively). Yield did not exhibit significant linear trends across the rows or columns and the column autocorrelation was moderate (0.37). In the Victoria canola trial a covariate describing crop lodging was fitted in the models for yield and HI without identifying a significant effect for either trait.

To summarise the impact of different types of insecticide-inputs used across all trials (regardless of pest management approach used) we conducted a MET analysis for yield (Table 5). We grouped the insecticide applications according to whether they were applied early season during crop establishment or later in the season, and if they were foliar sprays or applied as seed treatments. The results revealed a significant treatment effect for canola (*P* = 0.009) and no significant treatment effect for wheat (*P* = 0.104) (Table 5). The results for canola varied across treatments. The seed treatment alone and in combination with early season foliar spray showed the highest predicted yield, 2.4 and 2.8 t/ha respectively. For the other treatments (early spray alone, early spray in combination with late spray, and no treatment), the yield ranged from 0.25–1.0 t/ha. Still, one should take into account that early spray in combination with late spray treatment was only used at the WA sites, which were both very low yielding; therefore there is a confounding effect of treatment with climatic conditions. The results for wheat suggest that regardless of the pest management approach used, additional insecticide inputs did not increase crop yield. Still, there is an interesting pattern in the predicted yield means. The yield was 4 t/ha or higher for trials where seed treatment, snail baits or a combination of both were used (Table 5). The highest yield (4.8 t/ha) was observed for a treatment combination of snail baits and early season foliar spray, however snail baits were only used in SA so we cannot say how much influence snail baits alone would have. For trials where early season spray alone or in combination with late season spray was used or no treatment was applied, the predicted yield ranged from 3.5–3.9 t/ha.

**Table 5:** The effect of different insecticide inputs on crop yield analysed using a multi-environment approach.

Crop	Treatment#	Predicted mean yield (t/ha)	Standard error$
Canola	Early season foliar sprays	1.000	0.594
	Combined early and late season foliar sprays	0.253	0.839
	Combined early season foliar sprays and seed treatment	2.831	0.596
	Seed treatment only	2.392	0.486
	No insecticide applications	0.674	0.485
Wheat	Early season foliar sprays	3.533	0.450
	Combined early and late season foliar sprays	3.928	0.476
	Seed treatment only	3.994	0.476
	Snail baits	4.646	0.893
	Combined snail baits and early season foliar sprays	4.847	0.893
	Combined snail baits and seed treatment	4.453	0.893
	No insecticide applications	3.765	0.444

# See Table 1 for details about insecticide chemicals used.

$ Averaged SED for canola is 0.858 and for wheat 0.772.

We estimated the economic cost of insecticide inputs across the different treatments at each trial site (Table 6). Control treatments always had the lowest, or equal lowest, economic cost across all trials. The conventional treatments were more expensive than the low input treatments in nine trials. Only in one trial, WA1 in 2010, was the low input treatment ($61.61/ha) more expensive than the conventional treatment ($6.85/ha). The main expense for this low input treatment was the addition of a *Bt* spray (Table 1). When the economic costs were divided by the mean crop yield in each treatment, the conventional wheat plots had the highest average cost ($3.21/t/ha), followed by the low input ($2.38/t/ha) and control ($1.17/t/ha) treatments. In canola the low input treatment had the highest average costs ($61.97/t/ha) compared to the conventional ($24.35/t/ha) and control ($0.05/t/ha) treatments. However, the WA1 site heavily biased these estimates. When this trial was removed from the analysis, the low input treatment ($7.01/t/ha) was $17/t/ha less than the conventional treatment.

**Table 6:** Economic cost of insecticide inputs across the different treatments at each trial site, including the costs for the application of chemicals.

Crop	Site	Treatment		
		Control	Conventional	Low input
Canola	Victoria	0	13.67	0.70
	NSW	0.36	13.85	0.36
	SA	0.30	9.44	0.30
	WA1	0	6.85	61.61
	WA2	0	14.09	0
Wheat	Victoria	0	6.39	0
	NSW	0	5.98	0
	SA	21.80	30.75	30.20
	WA1	0	13.19	9.92
	WA2	0	6.64	0

All values expressed in AU$/ha.

## Discussion

A change of practice toward agroecosystem-based IPM requires three progressive steps [35], [36]. Firstly, an increased efficiency of pesticide inputs; secondly, input substitution with more benign chemicals or tactics; and thirdly, a system re-design that ensures the cropping landscape is less susceptible to pest-outbreaks. Our study addresses the first step in this process, by testing whether crops with greater inputs of insecticides experience higher crop yield. In theory, an application of an insecticide should lead to fewer pests, therefore less feeding damage to crop plants, and ultimately higher grain yields which would cancel out the economic cost of the insecticides (and the costs of applying chemicals). In reality this is a more complex process with some pest species able to withstand or avoid insecticide exposure [37], natural pest control services being lost as beneficial populations are reduced by the insecticide [38], secondary pest outbreaks occurring later in the season [39], plants being able to compensate for damage [40], and defend themselves against future damage [41], and differing costs of insecticide products and commodity prices. Given these complexities, the only way to adequately assess the effect of insecticides is to test them in as near to a commercial situation as possible, as we have done here using large replicated plots embedded in commercial cropping landscapes. What our empirical results show is that while insecticide use did impact the numbers of pests collected, we did not generally see higher yields in the conventionally managed plots (i.e. our hypothesis was not universally supported). Although there was clear evidence of feeding damage by arthropod pests in plots that were unsprayed, this did not translate into a lower yield compared to the conventional plots at the end of the season. Despite overall low abundance of beneficial arthropods we saw evidence of insecticide application reducing numbers of ground-dwelling and foliage-dwelling beneficials early in the season in Victoria and NSW canola. We suspect the mechanism underlying these patterns is a combination of greater tolerance to insecticides in particular pest species, lack of or loss of beneficial arthropods in some plots, and crop plants compensating for damage throughout the season.

Throughout our study there are few examples where yield loss in crops can be directly attributed to the activities of arthropod pests. If the results of these trials can be extended to commercial situations then there are opportunities for growers to reduce insecticide-inputs without risking yield loss. The yield loss observed within control plots at a single trial (WA1 canola) was due to the activities of mite pest species (*Penthaleus major* and *Halotydeus destructor*) early in the season (Figure C in File S4). This led to a significantly greater proportion of sucking damage and lower plant density in the control plots (Table 4) and ultimately contributed to the loss in yield (Fig. 2). However, it is important to note that yield potential in this trial was already greatly suppressed due to low rainfall (0.21 t/ha in the conventional and low input plots, compared with 0.09 t/ha in the control plots). Furthermore, there was no significant difference in yield between the conventional and low input plots suggesting the additional sprays within the conventional plots provided no yield benefit. The MET analysis results further support our conclusion that increased insecticide inputs does not necessarily lead to a yield gain (Table 5). Our results suggest that growers planting canola using insecticidal treated seed are likely to see some yield gain; however other insecticide inputs appear to be unnecessary in years with low-pest pressure. For growers planting wheat none of the insecticide inputs provided an economically justifiable yield gain in our trials.

Previous semi-field trials have shown that an IPM approach can lead to reduced pest populations, crop damage and higher yield in other broad-acre crops such as cotton and horticultural crops [15], [42]. Yet the application of IPM to grain crops in Australia has been limited. A similar statement was made by Wratten *et al.* [43] in 1995 regarding wheat in the UK, Netherlands, the USA and New Zealand. The low-input approach implemented in our study typically consisted of insecticide seed treatments, withholding insecticide applications if pest density was low, and replacing the conventional insecticides with a more target-specific or “low-risk” insecticide when pests reached a critical threshold. This is only one aspect of an IPM approach, ignoring cultural strategies implemented outside the season/field to encourage beneficial populations and reduce carry-over of pests [20] that would be difficult to include in a field-plot trial. The large size of our field plots (minimum of 2500 m^2^) allowed us to assess both direct and indirect insecticide effect patterns across the whole season for a variety of pest and beneficial species. These large plots are particularly useful when mobile arthropods are involved and for examining season-long effects of insecticide application [44]. Replicating the trials across five sites over two years allowed us to use these results to generalise across a wide area. However, repeating these trials in years with high pest pressure is required to assess thresholds at which the number and type of insecticides applied switches from providing crop protection to offering little advantage in terms of yield benefit. Alternatively, simulating high pest pressure by artificially infesting plots with pest species is possible (e.g., [45]), however undesirable if plots are located on commercial properties.

The incentive for grain growers to move towards a reduction in insecticide inputs is often based on short-term economic factors. In our study, we show that growers could potentially save money by altering their current insect pest management approaches in canola and wheat crops (Table 6). In four canola trials the cost of insecticide inputs was greatest in the conventional treatments, with an average of $12.67/ha. In comparison, the low input treatments cost an average of $0.34/ha. In the five wheat trials the cost of insecticides was greatest in the conventional treatments, with an average of $12.59/ha, compared to an average of $8.02/ha in the low input treatments. Despite the additional costs of the conventional farmer approach, there were no significant yield benefits over the low input approach in any trials. At one trial (WA1 canola), the economic cost of using insecticides in the low input plots was substantially greater (almost 10 times) compared with the conventional approach. This was principally due to the late season application of a selective insecticide, highlighting one of the largest barriers to widespread adoption of IPM among Australian grain growers – the high economic price of many selective chemicals. Although a reduction in insecticide inputs will lead to some direct cost savings, we anticipate that the additional costs associated with the implementation of IPM (e.g. the cost of selective insecticides, pest monitoring costs) will cancel out these savings. In the Australian context pesticide reduction strategies will be driven more by risk minimisation (also see [46]), sustainability and regulatory changes rather than direct economic benefits to the grower.

On only one occasion during our study were arthropod pest pressures above established economic spray thresholds (late season aphids in WA1 canola); so the low input plots were sprayed with insecticides considered low-risk to beneficials. Unfortunately, there are currently few soft insecticides, which are less disruptive to beneficial species registered for use in grain crops in Australia [22] and relatively little R&D investment into newer chemistries. It is encouraging then, that our results have shown, that in certain seasons with low pest pressure, grain growers can avoid insecticide spray applications altogether. However, for growers to be confident about abstaining from insecticide applications we must develop methods to forecast low pest pressure seasons in advance, and cost-effective in-season monitoring strategies that can be implemented across wide geographic areas. The results we have presented show that grain growers in a variety of agricultural landscapes in Australia can potentially farm with fewer pesticides. These growers have the potential to improve sustainability and environmental performance without a reduction in productivity. However this change of practice will not occur until greater emphasis is placed on developing new risk management tools and research into how IPM can be integrated in farm businesses.

## Supporting Information

Table S1 **Taxa that were included in the study and the functional groups into which they were classified.** Only Arthropoda were included (i.e. slugs, snails and earthworms ignored). ‘Pest’ included any common pest of grain crops across Australia, ‘Beneficial’ included natural enemies of these pests such as predators and parasitoids, ‘Other’ included other arthropods that could not be easily grouped into the previous categories. A summary value known as ‘all.arthropod’ included Pests, Beneficials and Others.
(DOCX)

Table S2 **Summary of the total number of pest and beneficial individuals captured using each sampling technique and included in the analyses.**
(DOCX)

File S1 **This file contains Figure A, B, and C.** Figure A, Pest and beneficial arthropods collected using pitfall traps (mean number per sample) at the Victoria trial site. At the trial site large plots (50 m×50 m minimum) were allocated to one of three pest management approaches; Conventional, Low Input, and Control with minimal insecticide inputs. Each dot represents the average of multiple samples collected within a plot. DAE = days after emergence, 0 and 2 indicates a pre-sow sample. Figure B, Pest and beneficial arthropods collected using sweep net sampling (mean number per sample) at the Victoria trial site. At the trial site large plots (50 m×50 m minimum) were allocated to one of three pest management approaches; Conventional, Low Input, and Control with minimal insecticide inputs. Each dot represents the average of multiple samples collected within a plot. DAE = days after emergence. Figure C, Pest and beneficial arthropods collected using vacuum sampler (number per sample) at the Victoria site. At the trial site large plots (50 m×50 m minimum) with three pest management approaches were assessed (treatment: conventional, low input, or control). Each dot represents the average of multiple samples collected within a plot. DAE = days after emergence, 0 indicates a pre-sow sample. One large outlier was removed from bottom RHS chart to improve clarity of the graphs.
(DOCX)

File S2 **This file contains Figure A, B, and C.** Figure A, Pest and beneficial arthropods collected using pitfall traps (mean number per sample) at the NSW trial site. At the trial site large plots (50 m×50 m minimum) were allocated to one of three pest management approaches; Conventional, Low Input, and Control with minimal insecticide inputs. Each dot represents the average of multiple samples collected within a plot. DAE = days after emergence, 0 and 2 indicates a pre-sow sample. Figure B, Pest and beneficial arthropods collected using sweep net sampling (mean number per sample) at the NSW trial site. At the trial site large plots (50 m×50 m minimum) were allocated to one of three pest management approaches; Conventional, Low Input, and Control with minimal insecticide inputs. Each dot represents the average of multiple samples collected within a plot. DAE = days after emergence. Figure C, Pest and beneficial arthropods collected using a vacuum sampler (number per sample) at the NSW trial site. At the trial site large plots (50 m×50 m minimum) were allocated to one of three pest management approaches (treatment: conventional, low input, or control). Each dot represents the average of multiple samples collected within a plot. DAE = days after emergence, 0 indicates a pre-sow sample.
(DOCX)

File S3 **This file contains Figure A, B, and C.** Figure A, Pest and beneficial arthropods collected using pitfall traps (mean number per sample) at the SA trial site. At the trial site large plots (50 m×50 m minimum) were allocated to one of three pest management approaches; Conventional, Low Input, and Control with minimal insecticide inputs. Each dot represents the average of multiple samples collected within a plot. DAE = days after emergence, 0 and 2 indicates a pre-sow sample. Figure B, Pest and beneficial arthropods collected using sweep net sampling (mean number per sample) at the SA trial site. At the trial site large plots (50 m×50 m minimum) were allocated to one of three pest management approaches; Conventional, Low Input, and Control with minimal insecticide inputs. Each dot represents the average of multiple samples collected within a plot. DAE = days after emergence. Figure C, Pest and beneficial arthropods collected using a vacuum sampler (number per sample) at the SA trial site. At the trial site large plots (50 m×50 m minimum) were allocated to one of three pest management approaches (treatment: conventional, low input, or control). Each dot represents the average of multiple samples collected within a plot. DAE = days after emergence, 0 indicates a pre-sow sample.
(DOCX)

File S4 **This file contains Figure A, B, and C.** Figure A, Pest and beneficial arthropods collected using pitfall traps (mean number per sample) at the WA1 trial site. At the trial site large plots (50 m×50 m minimum) were allocated to one of three pest management approaches; Conventional, Low Input, and Control with minimal insecticide inputs. Each dot represents the average of multiple samples collected within a plot. DAE = days after emergence, 0 and 2 indicates a pre-sow sample. In these pitfall traps all Collembola and Acari (mites) were excluded from the sorting. Figure B, Pest and beneficial arthropods collected using sweep net sampling (mean number per sample) at the WA1 trial site. At the trial site large plots (50 m×50 m minimum) were allocated to one of three pest management approaches; Conventional, Low Input, and Control with minimal insecticide inputs. Each dot represents the average of multiple samples collected within a plot. DAE = days after emergence. Figure C, Pest and beneficial arthropods collected using vacuum sampler (number per sample) at the WA1 site. At the trial site large plots (50 m×50 m minimum) with three pest management approaches were assessed (treatment: conventional, low input, or control). Each dot represents the average of multiple samples collected within a plot. DAE = days after emergence, 0 indicates a pre-sow sample. One large outlier was removed from bottom RHS chart to improve clarity of the graphs.
(DOCX)

File S5 **This file contains Figure A, B, and C.** Figure A, Pest and beneficial arthropods collected using pitfall traps (mean number per sample) at the WA2 trial site. At the trial site large plots (50 m×50 m minimum) were allocated to one of three pest management approaches; Conventional, Low Input, and Control with minimal insecticide inputs. Each dot represents the average of multiple samples collected within a plot. DAE = days after emergence, 0 and 2 indicates a pre-sow sample. In these pitfall traps all Collembola and Acari (mites) were excluded from the sorting. Figure B, Pest and beneficial arthropods collected using sweep net sampling (mean number per sample) at the WA2 trial site. At the trial site large plots (50 m×50 m minimum) were allocated to one of three pest management approaches; Conventional, Low Input, and Control with minimal insecticide inputs. Each dot represents the average of multiple samples collected within a plot. DAE = days after emergence. Figure C, Pest and beneficial arthropods collected using vacuum sampler (number per sample) at the WA2 site. At the trial site large plots (50 m×50 m minimum) with three pest management approaches were assessed (treatment: conventional, low input, or control). Each dot represents the average of multiple samples collected within a plot. DAE = days after emergence, 0 indicates a pre-sow sample. One large outlier was removed from each of the canola and wheat pest graphs to improve clarity.
(DOCX)
