# Supplementary material for: Reducing Insecticide Use in Broad-Acre Grains Production: An Australian Study
Source: PLoS One. 2014 Feb 19;9(2):e89119. doi: 10.1371/journal.pone.0089119 (PMC3929627; doi:10.1371/journal.pone.0089119)
Supplement: Table S1 — Taxa that were included in the study and the functional groups into which they were classified. Only Arthropoda were included (i.e. slugs, snails and earthworms ignored). ‘Pest’ included any common pest of grain crops across Australia, ‘Beneficial’ included natural enemies of these pests such as predators and parasitoids, ‘Other’ included other arthropods that could not be easily grouped into the previous categories. A summary value known as ‘all.arthropod’ included Pests, Beneficials and Others. (DOCX) [file pone.0089119.s001.docx]

**SUPPORTING INFORMATION Table S1.**

| Order/Class | Common name | Taxa | Functional group |
| --- | --- | --- | --- |
| Acari | redlegged earth mite | *Halotydeus destructor* | Pest |
|  | blue oat mite | *Penthaleus* spp. | Pest |
|  | balaustium mite | *Balaustium* spp. | Pest |
|  | bryobia mite | *Bryobia* spp. | Pest |
|  | other predatory mite (including snout mite) | other predatory mite | Beneficial |
|  | mesostig mite | Mesostigmata | Beneficial |
|  | anystid mite | Anystidae | Beneficial |
|  | Astigmata | Astigmata | Other |
|  | oribatid mite | Oribatid | Other |
| Araneae | spiders | Araneae | Beneficial |
|  | pseudoscorpion | Pseudoscorpionida | Other |
| Coleoptera | wireworm | Elateridae | Pest |
|  | wireworm larvae | Elateridae | Pest |
|  | false wireworm | Tenebrionidae | Pest |
|  | false wireworm larvae | Tenebrionidae | Pest |
|  | little pasture cockchafer | *Australaphodius frenchi* | Other |
|  | weevils | Curculionidae | Pest |
|  | carabids | Carabidae | Beneficial |
|  | carabid larvae | Carabidae larvae | Beneficial |
|  | rove beetle | Staphylinidae | Beneficial |
|  | ladybeetles | Coccinellidae | Beneficial |
|  | ladybeetle larvae | Coccinellidae | Beneficial |
|  | red & blue beetle | *Dicranolaius bellulus* | Beneficial |
|  | scavenger beetle | Lathrididae | Other |
|  | Pselaphidae | Pselaphidae | Other |
|  | scarabid | Scarabaeidae | Other |
|  | scarabid larvae | Scarabaeidae | Other |
|  | Anthicidae | Anthicidae | Other |
| Collembola | lucerne flea | *Sminthurus viridis* | Pest |
|  | scum Collembola | Other collembola | Other |
| Diptera | hoverfly adult | Syrphidae | Beneficial |
|  | hoverfly larvae | Syrphidae | Beneficial |
|  | robber fly | Asilidae | Beneficial |
|  | other fly | Diptera | Other |
| Hemiptera | aphid | Aphididae | Pest |
|  | leafhopper | Cicadellidae | Pest |
|  | rutherglen bug | Nysius vinitor | Pest |
|  | damsel bug | Nabidae | Beneficial |
|  | assassin bug | Reduviidae | Beneficial |
|  | minute pirate bug | *Orius* sp. | Beneficial |
|  | predatory shield bug | Pentatomidae | Beneficial |
|  | other bug | Hemiptera | Other |
| Hymenoptera | ants | Formicidae | Other |
|  | parasitoids | Hymenoptera | Beneficial |
|  | bees | Apoidea | Other |
| Lepidoptera | caterpillar | Lepidoptera | Pest |
|  | looper | Geometridae | Pest |
|  | moth or butterfly | Lepidoptera | Pest |
|  | lacewing | Neuroptera | Beneficial |
|  | dragonfly | Odonata | Beneficial |
|  | thrips | Thysanoptera | Pest |
|  | slaters | Isopoda | Pest |
|  | cricket or grasshopper | Orthoptera | Pest |
|  | Australian plague locust | *Chortoicetes terminifera* | Pest |
|  | European earwig | *Forficula auricularia* | Pest |
|  | native earwig | *Labidura truncata* | Beneficial |
|  | millipede | Diplopoda | Pest |
|  | centipede | Chilopoda | Beneficial |
|  | booklice | Psocoptera | Other |
|  | cockroach | Blattodea | Other |
