# Supplementary material for: Reducing Insecticide Use in Broad-Acre Grains Production: An Australian Study
Source: PLoS One. 2014 Feb 19;9(2):e89119. doi: 10.1371/journal.pone.0089119 (PMC3929627; doi:10.1371/journal.pone.0089119)
Supplement: Table S2 — Summary of the total number of pest and beneficial individuals captured using each sampling technique and included in the analyses. (DOCX) [file pone.0089119.s002.docx]

**SUPPORTING INFORMATION Table S2**

|  | Sampling technique | Pests | Beneficials |
| --- | --- | --- | --- |
| Canola | Pitfall | 32695 | 12796 |
|  | Vacuum | 18502 | 2634 |
|  | Sweep | 67196 | 17825 |
| Wheat | Pitfall | 14213 | 14960 |
|  | Vacuum | 5496 | 5940 |
|  | Sweep | 96905 | 9707 |
| Total |  | 235007 | 63862 |
