# Supplementary material for: Reducing Insecticide Use in Broad-Acre Grains Production: An Australian Study
Source: PLoS One. 2014 Feb 19;9(2):e89119. doi: 10.1371/journal.pone.0089119 (PMC3929627; doi:10.1371/journal.pone.0089119)
Supplement: File S1 — This file contains Figure A, B, and C. Figure A, Pest and beneficial arthropods collected using pitfall traps (mean number per sample) at the Victoria trial site. At the trial site large plots (50 m×50 m minimum) were allocated to one of three pest management approaches; Conventional, Low Input, and Control with minimal insecticide inputs. Each dot represents the average of multiple samples collected within a plot. DAE = days after emergence, 0 and 2 indicates a pre-sow sample. Figure B, Pest and beneficial arthropods collected using sweep net sampling (mean number per sample) at the Victoria trial site. At the trial site large plots (50 m×50 m minimum) were allocated to one of three pest management approaches; Conventional, Low Input, and Control with minimal insecticide inputs. Each dot represents the average of multiple samples collected within a plot. DAE = days after emergence. Figure C, Pest and beneficial arthropods collected using vacuum sampler (number per sample) at the Victoria site. At the trial site large plots (50 m×50 m minimum) with three pest management approaches were assessed (treatment: conventional, low input, or control). Each dot represents the average of multiple samples collected within a plot. DAE = days after emergence, 0 indicates a pre-sow sample. One large outlier was removed from bottom RHS chart to improve clarity of the graphs. (DOCX) [file pone.0089119.s003.docx]

**SUPPORTING INFORMATION: PEST AND BENEFICIAL ARTHROPOD ABUNDANCE ACROSS TIME FOR EACH SAMPLING TECHNIQUE**

**Victoria site**

**
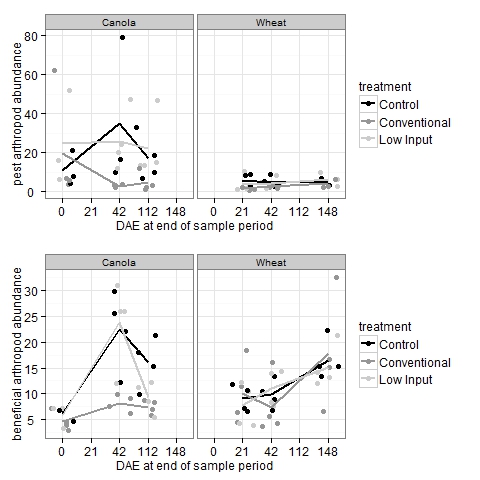
**

**Figure A.**

**
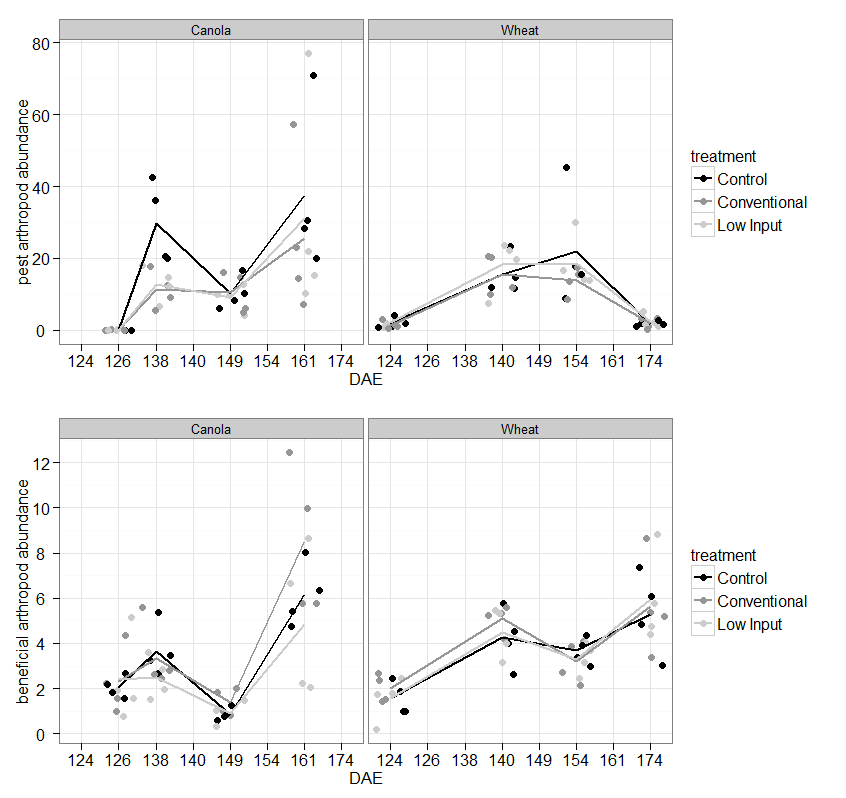
**

**Figure B.**

**
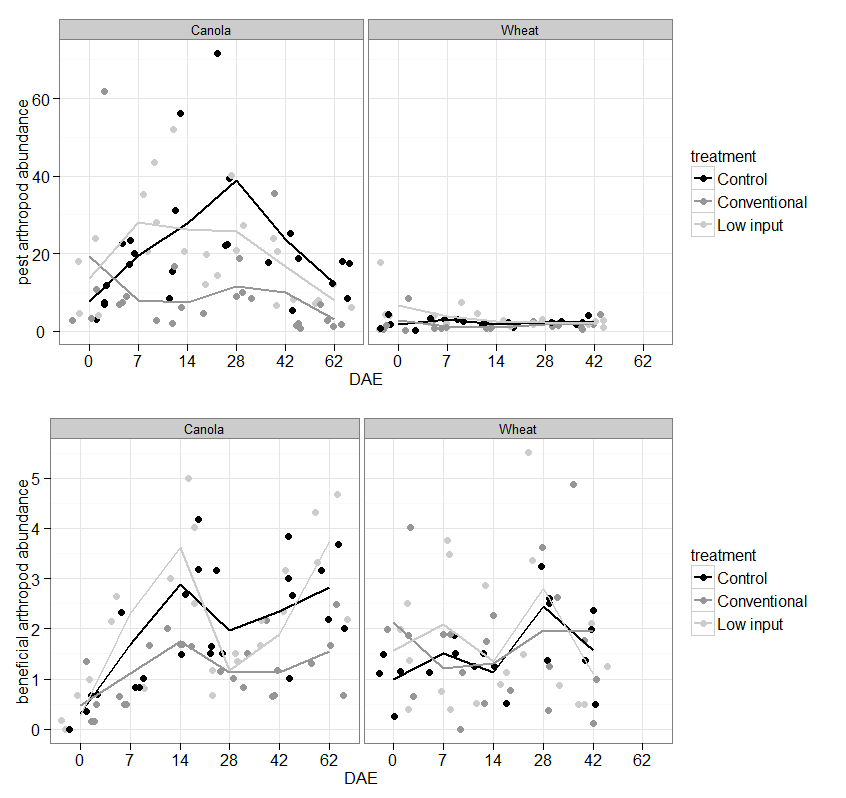
**

**Figure C.**
