# Supplementary material for: Reducing Insecticide Use in Broad-Acre Grains Production: An Australian Study
Source: PLoS One. 2014 Feb 19;9(2):e89119. doi: 10.1371/journal.pone.0089119 (PMC3929627; doi:10.1371/journal.pone.0089119)
Supplement: File S5 — This file contains Figure A, B, and C. Figure A, Pest and beneficial arthropods collected using pitfall traps (mean number per sample) at the WA2 trial site. At the trial site large plots (50 m×50 m minimum) were allocated to one of three pest management approaches; Conventional, Low Input, and Control with minimal insecticide inputs. Each dot represents the average of multiple samples collected within a plot. DAE = days after emergence, 0 and 2 indicates a pre-sow sample. In these pitfall traps all Collembola and Acari (mites) were excluded from the sorting. Figure B, Pest and beneficial arthropods collected using sweep net sampling (mean number per sample) at the WA2 trial site. At the trial site large plots (50 m×50 m minimum) were allocated to one of three pest management approaches; Conventional, Low Input, and Control with minimal insecticide inputs. Each dot represents the average of multiple samples collected within a plot. DAE = days after emergence. Figure C, Pest and beneficial arthropods collected using vacuum sampler (number per sample) at the WA2 site. At the trial site large plots (50 m×50 m minimum) with three pest management approaches were assessed (treatment: conventional, low input, or control). Each dot represents the average of multiple samples collected within a plot. DAE = days after emergence, 0 indicates a pre-sow sample. One large outlier was removed from each of the canola and wheat pest graphs to improve clarity. (DOCX) [file pone.0089119.s007.docx]

**SUPPORTING INFORMATION: PEST AND BENEFICIAL ARTHROPOD ABUNDANCE ACROSS TIME FOR EACH SAMPLING TECHNIQUE**

**WA2 site**


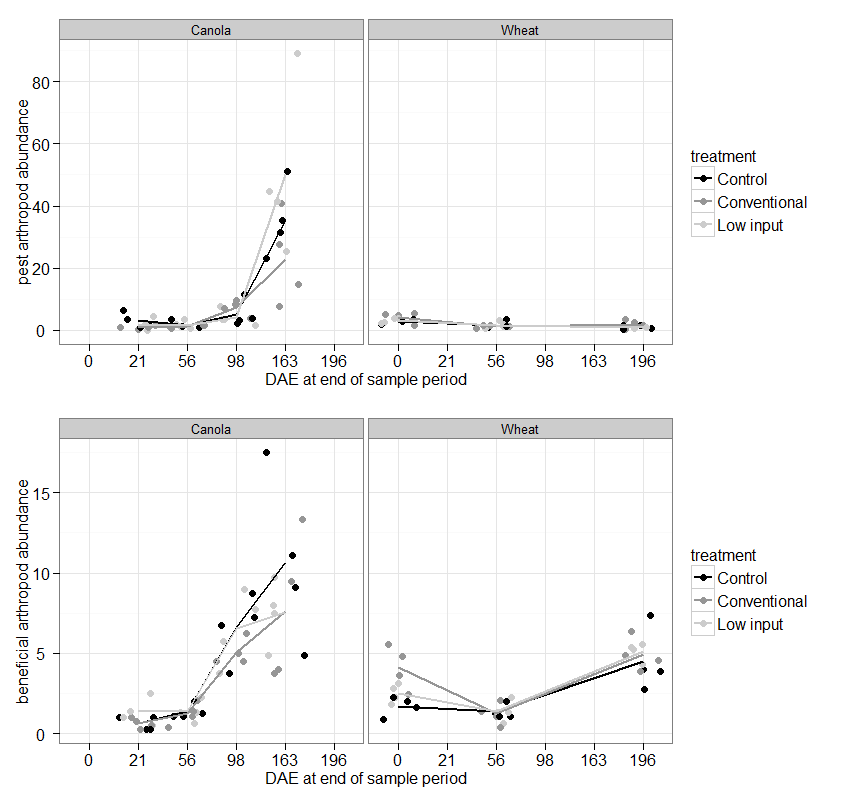


**Figure A.**

**
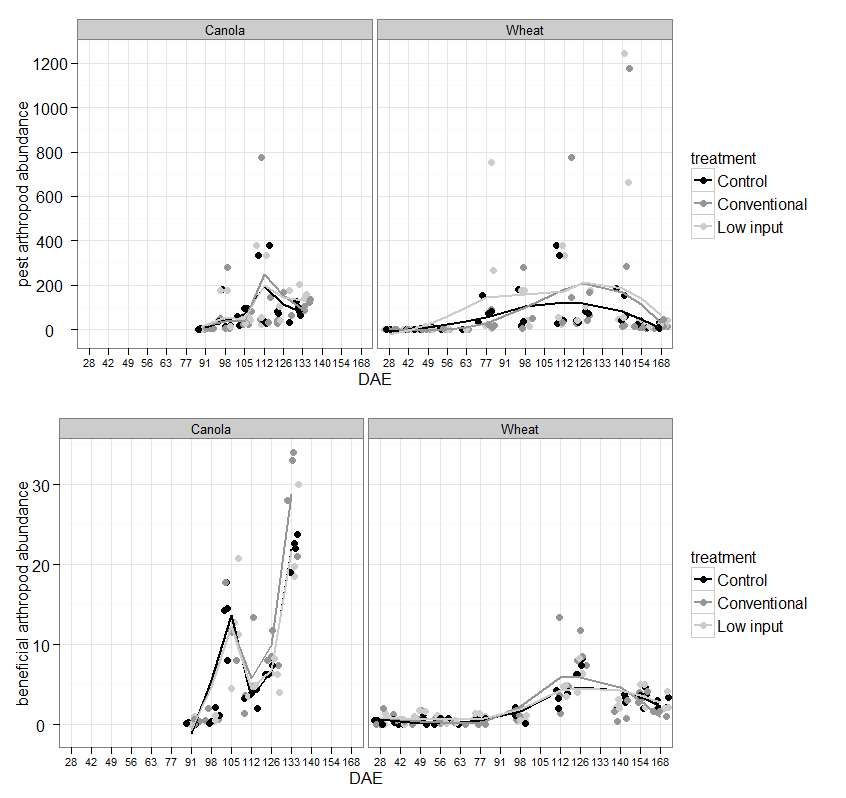
 Figure B.**

**
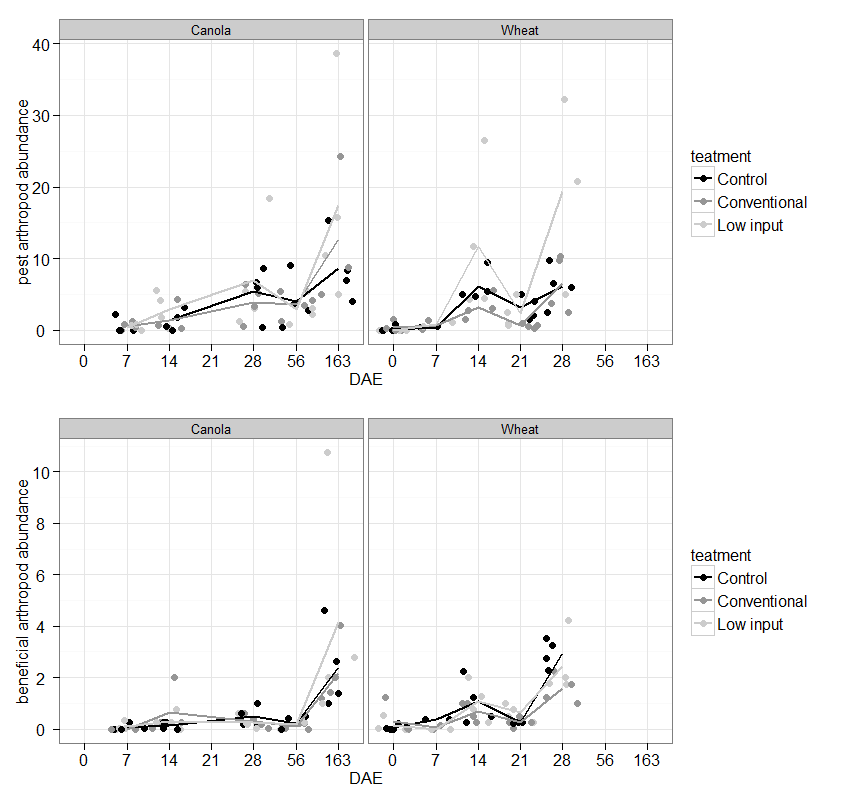
**

**Figure C.**
